# Comprehensive analysis of CCCH-type zinc finger family genes facilitates functional gene discovery and reflects recent allopolyploidization event in tetraploid switchgrass

**DOI:** 10.1186/s12864-015-1328-4

**Published:** 2015-02-25

**Authors:** Shaoxun Yuan, Bin Xu, Jing Zhang, Zheni Xie, Qiang Cheng, Zhimin Yang, Qingsheng Cai, Bingru Huang

**Affiliations:** College of Life Science, Nanjing Agricultural University, Nanjing, 210095 PR China; College of Agro-grassland Science, Nanjing Agricultural University, Nanjing, 210095 PR China; Jiangsu Key Laboratory for Poplar Germplasm Enhancement and Variety Improvement, Nanjing Forestry University, Nanjing, 210037 PR China; Department of Plant Biology and Pathology, Rutgers, the State University of New Jersey, New Brunswick, NJ 08901 USA

**Keywords:** *Panicum virgatum*, C3H, Evolution, Polyploidy, Stress, Development

## Abstract

**Background:**

In recent years, dozens of Arabidopsis and rice CCCH-type zinc finger genes have been functionally studied, many of which confer important traits, such as abiotic and biotic stress tolerance, delayed leaf senescence and improved plant architecture. Switchgrass (*Panicum virgatum*) is an important bioenergy crop. Identification of agronomically important genes and/or loci is an important step for switchgrass molecular breeding. Annotating switchgrass *CCCH* genes using translational genomics methods will help further the goal of understanding switchgrass genetics and creating improved varieties.

**Results:**

Taking advantage of the publicly-available switchgrass genomic and transcriptomic databases, we carried out a comprehensive analysis of switchgrass *CCCH* genes (*PvC3H*s). A total of 103 *PvC3H*s were identified and divided into 21 clades according to phylogenetic analysis. Genes in the same clade shared similar gene structure and conserved motifs. Chromosomal location analysis showed that most of the duplicated *PvC3H* gene pairs are in homeologous chromosomes. Evolution analysis of 19 selected *PvC3H* pairs showed that 42.1% of them were under diversifying selection. Expression atlas of the 103 *PvC3H*s in 21 different organs, tissues and developmental stages revealed genes with higher expression levels in lignified cells, vascular cells, or reproductive tissues/organs, suggesting the potential function of these genes in development. We also found that eight *PvC3H*s in Clade-XIV were orthologous to ABA- or stress- responsive *CCCH* genes in Arabidopsis and rice with functions annotated. Promoter and qRT-PCR analyses of Clade-XIV *PvC3H*s showed that these eight genes were all responsive to ABA and various stresses.

**Conclusions:**

Genome-wide analysis of *PvC3H*s confirmed the recent allopolyploidization event of tetraploid switchgrass from two closely-related diploid progenitors. The short time window after the polyploidization event allowed the existence of a large number of *PvC3H* genes with a high positive selection pressure onto them. The homeologous pairs of *PvC3H*s may contribute to the heterosis of switchgrass and its wide adaptation in different ecological niches. Phylogenetic and gene expression analyses provide informative clues for discovering *PvC3H* genes in some functional categories. Particularly, eight *PvC3Hs* in Clade-XIV were found involved in stress responses. This information provides a foundation for functional studies of these genes in the future.

**Electronic supplementary material:**

The online version of this article (doi:10.1186/s12864-015-1328-4) contains supplementary material, which is available to authorized users.

## Background

Zinc finger proteins, a large family in eukaryotes, make tandem contacts with their target molecules, such as the metal ion zinc, DNA, RNA, proteins and lipids, through their Zinc finger (Znf) motifs [1]. Their binding properties depend on the Znf domain’s sequence, the number of Znf domains and the protein’s higher-order structures [1]. The CCCHs, a unique subfamily of Znf proteins, feature a characteristic motif(s) comprising of three Cys and one His residues [2]. The number of CCCH proteins varies across diploid plant species, from 34 in *Medicago truncatula* [3] to 91 in poplar tree (*Populus trichocarpa*) [4]. So far, most identified CCCH proteins in plant species have one to six CCCH motifs [3-7]. The consensus sequence of the CCCH motif can be further classified according to the number of amino acid between the Cys and His residues in the CCCH motif, and most CCCH motifs contains C-X_4–15_-C-X_4–6_-C-X_3–4_-H sequence (X for any amino acid) [6].

In planta, *CCCH* genes play pivotal roles in cell fate specification and hormone-regulated stress responses. Till now, most reported plant *CCCH* genes were identified through differential expression analyses (e.g. *AtPEI1*, *AtTZF1, OsDOS*, and *GhZFP1*) or forward genetics approaches (e.g. *AtHUA1* and *AtSZF1/2*). For example, *AtPEI1*, an embryo-specific *CCCH* gene that is indispensable for heart-stage embryo formation, was first isolated using a virtual subtraction method from the cDNA library of Arabidopsis embryos [8]. Using a differential hybridization screening, a cotton *CCCH* gene, *GhZFP1*, was isolated, which functions through interacting with a dehydration protein and a pathogenesis-related protein to positively regulate both salt tolerance and disease resistance [9]. Through microarray studies, *AtTZF1* [10] and *OsDOS* [11] were identified as differentially expressed genes to sugar response or during pollination, respectively. Overexpressing *AtTZF1* resulted in compact statured plants, late flowering and higher stress-tolerance through positively regulating abscisic acid (ABA)/sugar responses and negatively regulates gibberellic acid (GA) responses [10]; while overexpressing *OsDOS* in rice produced a marked delay of leaf senescence primarily through negatively regulating the jasmonic acid (JA) pathway [11]. Through the screening of developmental or salt-sensitive mutants and a map-based cloning approach (forward genetics), Arabidopsis genes *AtHUA1* and *AtSZF1*/*2* were identified and cloned [12]. *AtHUA1* acts in floral morphogenesis by specifically processing *AGAMOUS* pre-mRNAs [12,13]; while *AtSZF1* and *AtSZF2* negatively regulate the expression of many salt-responsive genes and positively modulate the tolerance of Arabidopsis to salt stress [13].

Homologous gene analysis is another useful method to discover important genetic components. For example, *CCCH* genes *OsTZF1* and *AtTZF2/3/4/5/6* were identified in this way. *OsTZF1* was isolated as the rice ortholog to AtTZF1 [14]. Expression of *OsTZF1* is induced by drought, salt, hydrogen peroxide, as well as ABA, JA and salicylic acid (SA) [14]. Overexpression of *OsTZF1* in transgenic rice has delayed seed germination, delayed leaf senescence, and enhanced tolerances to drought and salt stress, through regulating the downstream genes’ pre-mRNA stability by directly binding to U-rich regions in the 3’ -UTRs [14]. Arabidopsis genes, *AtTZF2/3/4/5/6*, were studied as close paralogous genes to *AtTZF1* [15,16]*.* The expression patterns of *AtTZF2/3* are similar to *AtTZF1* and transcripts of these two genes can be found in various vegetative tissues and in flowers. Similar to *OsTZF1*, overexpression of *AtTZF2/3* caused delayed senescence, enhanced longevity, and larger plants at the mature stage [15]. Unlike those of *AtTZF1/2/3*, expression of *AtTZF4/5/6* are specific to seeds [16]. The expression levels of *AtTZF4/5/6* decline during seed imbibition, and are up-regulated by ABA and down-regulated by GA. Mutant analysis showed that *AtTZF4/5/6* are negative regulators for light- and GA-mediated seed germination responses by controlling genes critical for ABA and GA responses [16].

Switchgrass (*Panicum virgatum* L.) is a warm-season C_4_ perennial grass used for bioenergy and animal feedstock [17,18]. To avoid competing with food crops for arable field, a large proportion of switchgrass fields will be located on marginal lands where various abiotic stresses, such as salt, drought, and extreme temperatures, limit plant growth. Translating the knowledge gained from the study of model plant species, such as Arabidopsis, into crop species has contributed to improving important agronomic problems in major food crops [19,20]. For example, an Arabidopsis gene, *Sodium Proton Exchanger 1* (*AtNHX1*), was identified as a key regulator of salt tolerance in Arabidopsis. When *AtNHX1* was overexpressed in *Brassica napus*, tomato, and rice, all of the transgenic plants acquired a significant improvement in salt tolerance [20].

*CCCH* genes have great potential for plant genetic improvement. Mutant selection, differential gene expression, and homologous gene analyses are three classical approaches to identify important *CCCH* genes as described previously. However, since switchgrass is a self-incompatible grass with a complex allotetraploid genome, it is difficult to pinpoint important genes/loci in switchgrass using forward genetic tools (e.g. mutant selection and map-based cloning). Comprehensive gene family analysis combined with translational genomics provides an unprecedented opportunity to predict potential functions of *CCCH* genes. For example, Wang et al. predicted that certain subfamilies of the CCCH proteins in Arabidopsis were involved in stress tolerances, and showed that the subfamily IX gene members responded to salt, ABA, drought, and cold stresses [6]. Also, Peng et al. showed that *CCCH* subfamily I genes in maize were responsive to ABA and drought stimuli [7]. It is rational to adopt this strategy to conduct genome-wide comprehensive analysis on switchgrass *CCCH* genes as well.

The latest version of the switchgrass genome database (*Panicum virgatum* v1.1, DOE-JGI) includes 15× sequence coverage of the genome with about 6.5× from long linear reads [21]; in addition, over 93% of the protein-coding genes have been annotated [22]. This genomic dataset together with the transcriptomic databases (pviUTs & PviGEA) [23,24] provide us with a quality framework to address questions of biological significance from the perspective of genetic components. Here we make use of the publicly available switchgrass genomic [21] and transcriptomic [23,24] databases to systematically analyze *CCCH* gene family and to identify candidate genes contributing to plant development and stress tolerance in switchgrass.

## Results

### Identification of CCCH proteins in switchgrass

The newly released genome database of “*Panicum virgatum* v1.1, DOE-JGI” [21] was used in this study. After extensive searches of the database with the Hidden Markov Model (HMM) file PF00642 and manual analysis to remove the false positive and redundant genes, a total of 103 switchgrass *CCCH* genes were identified and designated as *PvC3H1* to *PvC3H103* (Additional file 1). The complex allotetraploid genetic background of lowland switchgrass makes it a great challenge to assemble the two sets of heterozygous genomes and reach chromosome-scale contiguity [25]. In this study, we obtained complete sequences of 94 *PvC3H*s from the genome database (Phytozome) and another 6 full length *PvC3H*s from the transcriptome data (PviUTs) [23,24] by aligning and joining overlapping transcripts. Three *PvC3H*s’ sequences were incomplete (Additional file 1). The deduced full lengths of PvC3H proteins ranged from 121aa to 1358aa, among which only two were more than 1000aa in length (Additional file 1).

**Figure 1:**
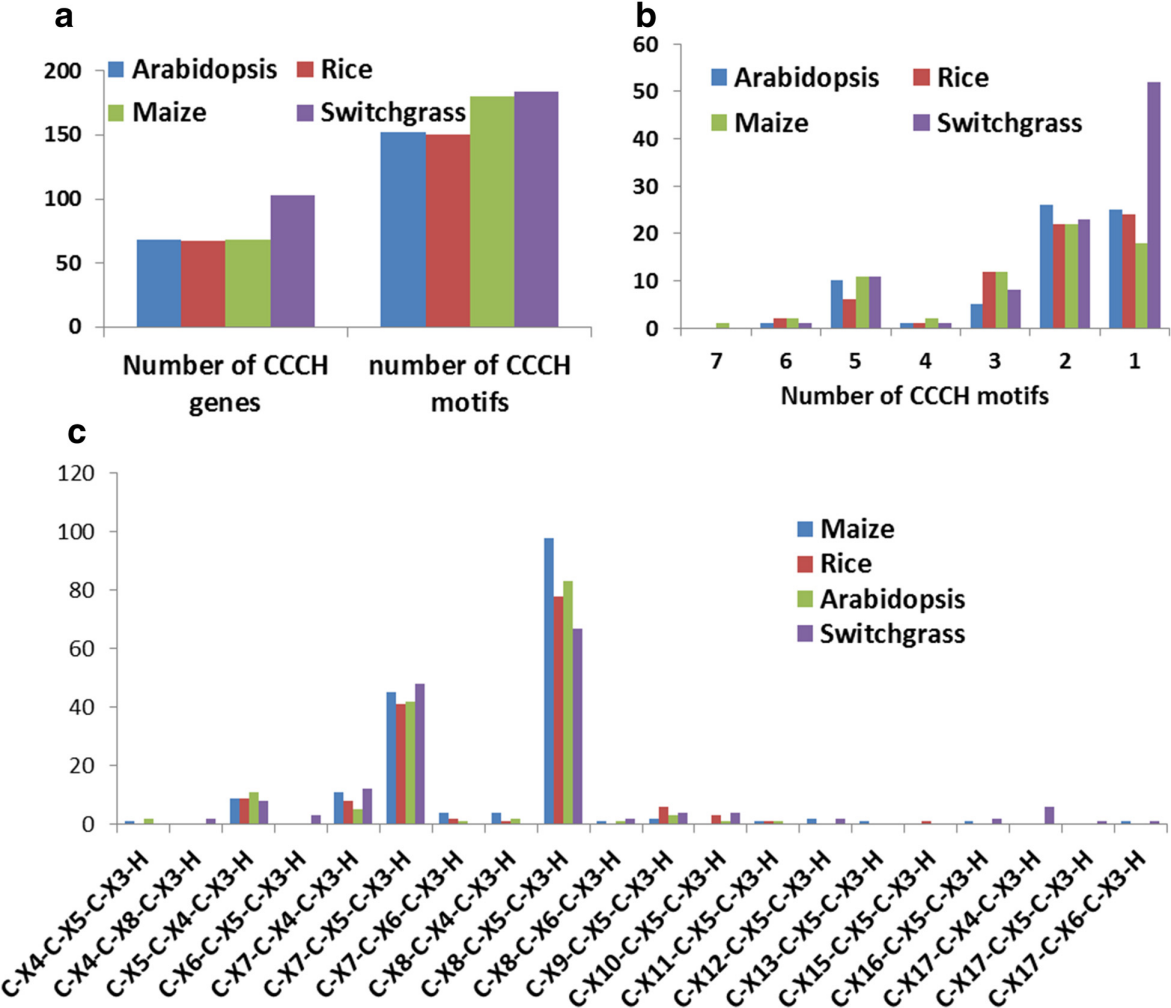
**Characterization of CCCH proteins in switchgrass, maize, rice and Arabidopsis. (a)** Number of CCCH proteins and Zf-CCCH motifs in the four plant species. **(b)** Number of CCCH motifs per protein. **(c)** Number of each type of Zf-CCCH motifs in the four plant species.

The number of CCCH motifs in PvC3H proteins was calculated using the Pfam and SMART programs. As shown in Figure 1, there were a total of 202 CCCH motifs in PvC3H proteins, which was comparable to that of maize (180) and higher than Rice (150) and Arabidopsis (152) (Figure 1a). The PvC3H proteins had one to six CCCH motifs per protein (Figure 1b). Notably, the number of PvC3Hs with only one CCCH motif (53) was much higher than that of Arabidopsis (18), rice (24) and maize (25).

The classical CCCH motif was defined as C-X_4–15_-C-X_4–6_-C-X_3_-H [6]. According to the spacer numbers in-between the Cys residues, the CCCH motif could be classified into different patterns. In switchgrass and the other three plant species, the most common CCCH motifs had patterns of C-X_8_-C-X_5_-C-X_3_-H and C-X_7_-C-X_5_-C-X_3_-H. Rare CCCH motif patterns were also found among PvC3Hs: two had motifs of C-X_16_-C-X_5_-C-X_3_-H, six had C-X_17_-C-X_4_-C-X_3_-H, one had C-X_17_-C-X_5_-C-X_3_-H, and one had C-X_17_-C-X_6_-C-X_3_-H (Figure 1c). Notably, the motif pattern of C-X_17_-C-X_4–6_-C-X_3_-H was only found once in a maize CCCH protein, *ZmC3H17* [7].

The sequence logos of the four most common types of CCCH motifs were compared between switchgrass, maize, rice and Arabidopsis. As illustrated in Additional file 2, the two most common CCCH motif patterns had different sequence logos. Within each motif pattern, sequence logos were found to be similar across the four plant species, and the degree of similarity between the four plant species was consistent with their phylogenetic relationships (Additional file 2).

### Phylogenetic and structural analyses

**Figure 2:**
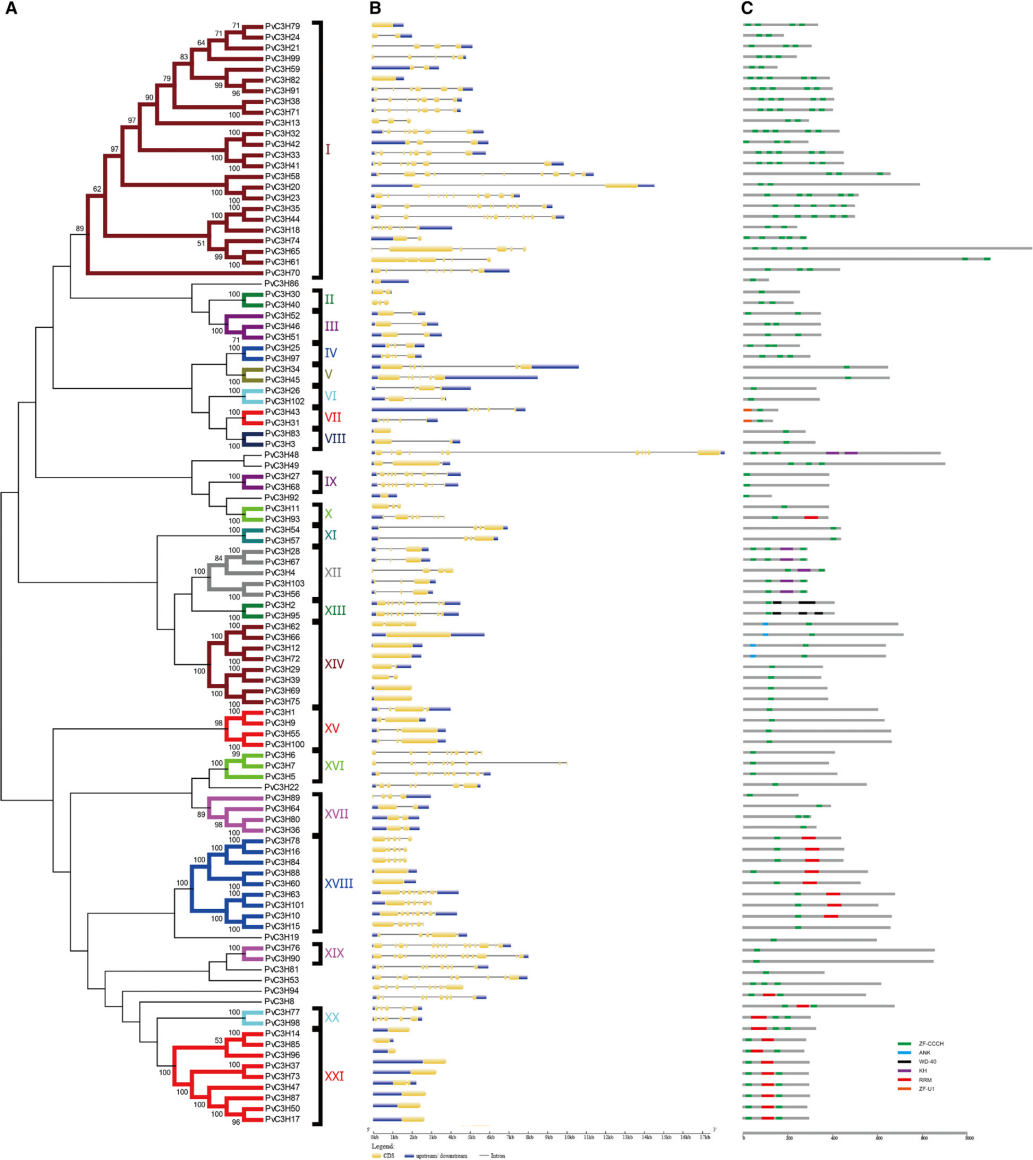
**Evolutionary relationships (A), gene structures (B) and functional motifs (C) of PvC3Hs.** The evolutionary history was inferred using the N-J method [26]. The optimal tree with the sum of branch length = 24.37 is shown. Bootstrap values of 1,000 replications were executed [27], and only results with a score above 50 are shown at each node. The evolutionary distances were computed using the p-distance method [28] and are in the units of the number of amino acid differences per site. Evolutionary analyses were conducted in MEGA6 [29].

We constructed neighbor-joining (N-J) phylogenetic trees to illustrate the evolutionary relationships between the PvC3Hs (Figure 2a) and between all identified CCCH proteins in switchgrass, maize, rice and Arabidopsis (Additional file 3). We determined the relationships (clades) between proteins and identified a total of 21 clades including 94 PvC3Hs with the rest nine PvC3Hs as singletons based on bootstrap value >50 (Figure 2a). PvC3Hs within the same clade shared similar exon-intron structures of their encoding genes (Figure 2b) and similar numbers and distributions of functional motifs (Figure 2c). Despite the variable lengths and sequences of introns, the number of introns and the lengths of individual exons were highly similar across the PvC3Hs within the same clade. Conserved exon-intron structures and motif distribution orders across the PvC3Hs in each clade strongly supported the reliability of the phylogenetic tree. Taking Clade-XX & -XXI PvC3Hs as examples, proteins in Clade-XX had one RNA-Recognition Motif (RRM) near to the N- terminal and two CCCH motifs after the RRM; while most Clade-XXI proteins had two CCCH motifs and one RRM in-between.

CCCH proteins have been found to regulate post-transcriptional modification of downstream target pre-mRNAs [30,31], interacting with different proteins (e.g. GhZFP1) [9], or transcriptionally activating/repressing target genes (e.g. AtHUA1, AtPEI & OsLIC1) [8,12,32]. Functional motifs found among PvC3Hs include RRMs and K homolog domains (KH) that are involved in RNA processing, and Ankyrin repeats (Ank), WD40 repeats (WD40) and RING motifs that are involved in protein-protein interactions or multi-protein complex assembly (Figure 2). Specifically, PvC3Hs in clades-XVII, −XX, and -XXI had one or two RRM motifs (Figure 2) suggesting PvC3Hs within these three clades could have conserved roles through processing downstream target mRNAs.

**Figure 3:**
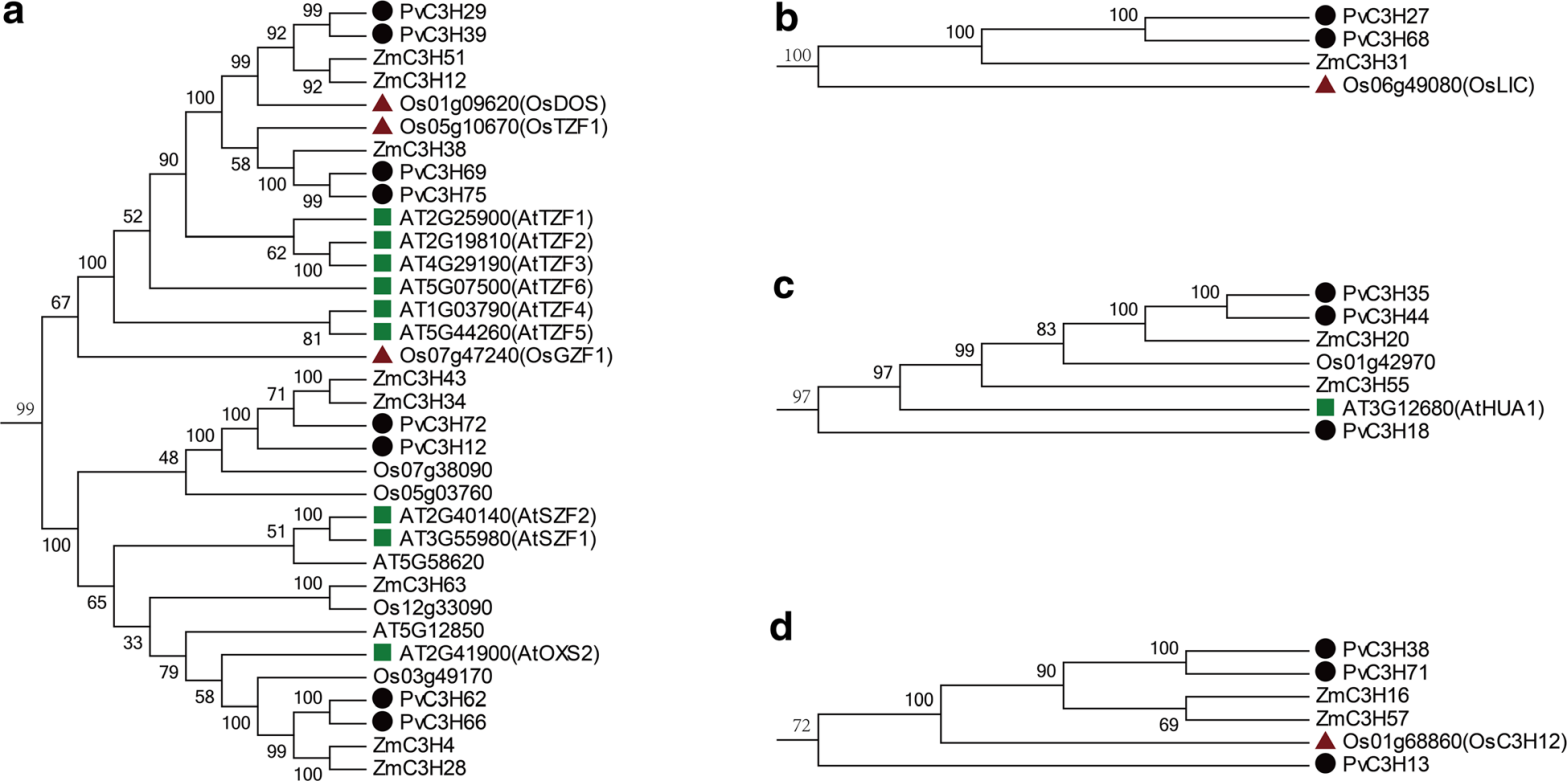
**Phylogenetic relationships pinpoints the PvC3H orthologs to functional-annotated CCCH proteins in Arabidopsis and rice. (a)** Clade-XIV *PvC3H* genes were orthologous to ABA- or stress-responsive *CCCH* genes. **(b)** *PvC3H27/68* in Clade-IX were orthologous to *OsLIC*. **(c)** *PvC3H35*/*44* in Clade I were orthologous to *AtHUA1*. **(d)** *PvC3H38*/*71* in Clade-I were orthologous to *OsC3H12*.

The CCCH families in Arabidopsis, rice, maize and switchgrass were further compared (Additional file 3). Most *CCCH* genes were clustered with their paralogs in the same species. Except for a few species-specific genes, most rice and maize *CCCH* genes had one or a pair of orthologs in switchgrass (Figure 3 and Additional file 4). We attempted to find PvC3Hs which were orthologous to functionally-annotated Arabidopsis and rice CCCH proteins, and found that ABA- or stress-responsive *CCCH* genes, such as *OsTZF1* [14], *OsDOS* [11], *AtTZF1*/*2*/*3* [10,15], and *AtSZF1*/*2* [13] were orthologous to the Clade-XIV *PvC3H* genes, *OsC3H12* [33] was orthologous to *PvC3H38*/*71* in Clade-I, *AtHUA1* [12] was orthologous to *PvC3H35*/*44* in Clade I, and *OsLIC* [32] was orthologous to *PvC3H27/68* in Clade-IX (Figure 3 and Additional file 4).

### Chromosomal locations and duplications in homeologous chromosomes

**Figure 4:**
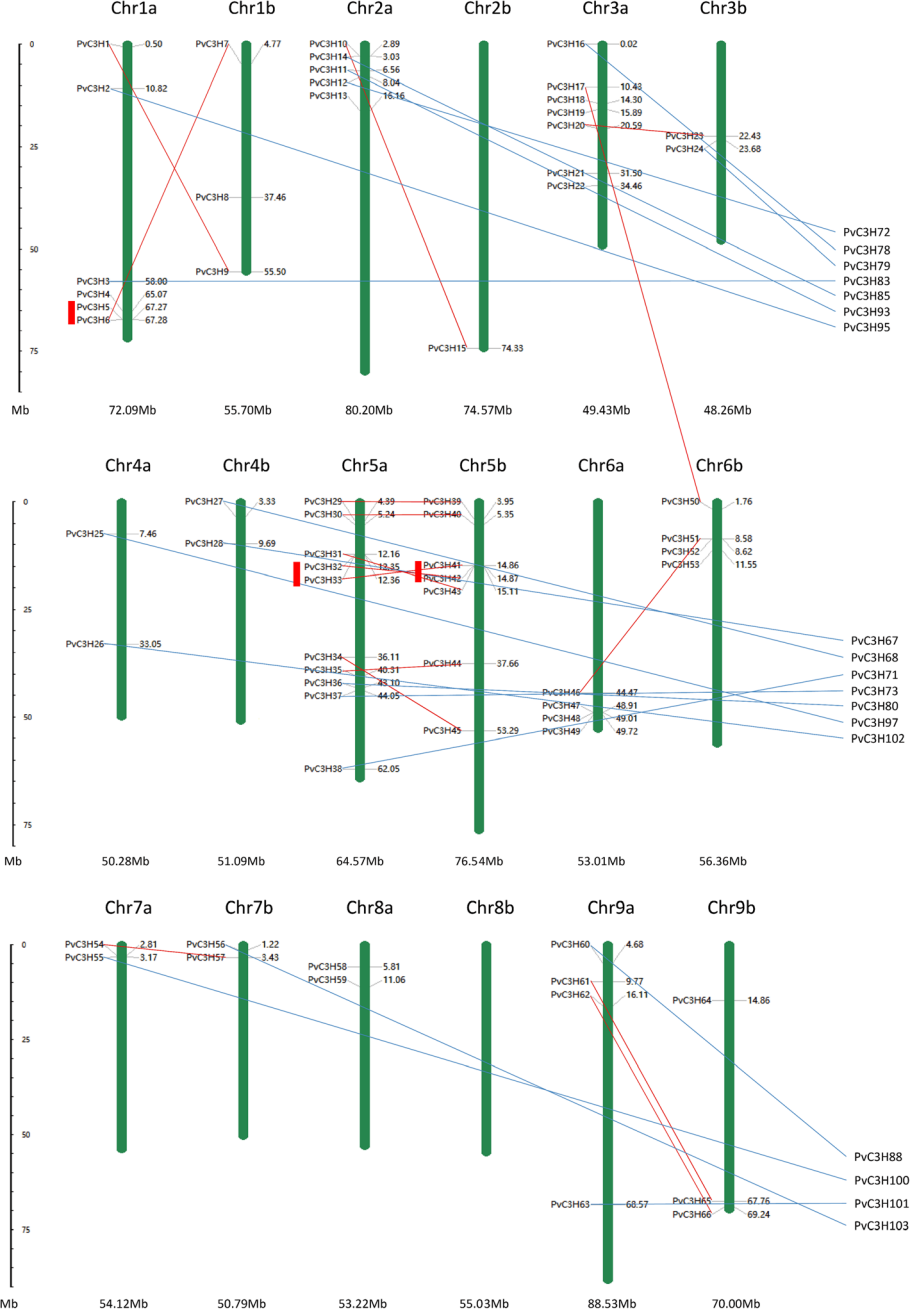
**Chromosomal locations of 66** ***PvC3H*** **s.** For those with unknown chr locations, we listed them on the right side of the figure. Duplications caused by allotetraploidy were connected by dashed red lines (between genes with known chr. locations) or blue lines. Tandem duplications were marked by red bars.

According to Okada et al. [34], allotetraploid switchgrass had two subgenomes, designated as A and B. In this study, chromosomal (Chr.) localizations of 66 *PvC3H*s were found in the two subgenomes which were unevenly distributed on 18 chromosomes of nine homeologous pairs. According to the phylogenetic tree (Figure 4), we linked the paralogous pairs of *PvC3H*s, and found a total of 16 pairs of paralogous *PvC3Hs* with defined chr. locations (red lined pairs in Figure 4). Most of these 16 pairs were in homeologous chromosomes with only one exception (*PvC3H17 & PvC3H50*). Tandem gene duplication was defined as paralogous genes physically linked in tandem with less than five gene loci in-between. With that definition, three tandem duplications were found: *PvC3H5/6* on Chr1a, *PvC3H32/33* on Chr5a, and *PvC3H41/42* on Chr5b. Among maize CCCH proteins, two tandem duplications were also found (*ZmC3H46*/*47*, *ZmC3H13*/*14*) [7]. We checked whether these tandem gene duplications were within large microsyntenous regions between switchgrass and maize. As illustrated in the Additional file 5, *PvC3H29/32/33* on switchgrass Chr5a and *ZmC3H12/13/14* on maize Chr3, and *PvC3H41/42/43* on switchgrass Chr5b and *ZmC3H46/47/49* on maize Chr8 were two syntenic gene sets. The conservation and micro-colinearity of *CCCH* genes suggest a common origin of these genes.

Fixation of advantageous mutations usually leads to evolutionary innovations and species divergences (so called ‘positive or diversifying selection’), while the removal of deleterious alleles or mutations maintains the natural fitness of species (so called ‘negative or purifying selection’). Here, we adopted phylogenetic comparison of synonymous and nonsynonymous substitution rates to tell the selection mode between *PvC3H* paralogous genes (Table 1). A total of 19 paralogous pairs [bootstrap value >95 in the phylogenetic tree (Figure 2)] of *PvC3Hs* with defined chr locations were compared, among which 16 pairs were caused by allotetraploidy (15 pairs of homeologous genes and 1 pair on non-homeologous chromosomes), and 3 pairs of tandem duplicated genes. According to the Ka/Ks ratio, 42.1% (8 out of 19) *CCCH* gene pairs were under diversifying selection. This percentage is much higher than that found in maize (11.8%) [7]. Furthermore, homeologous genes’ divergence time was estimated to be 3–11.6 million years ago (Mya); three tandem duplicated genes diverged 12.5-22.1 Mya; while the pair (*PvC3H17*/*50*) on non-homeologous chromosomes diverged 18.2 Mya. It was estimated that the two diploid progenitors of tetraploid switchgrass diverged ~2 Mya [35]. Therefore, the tandem duplicated pairs happened before the divergence of the progenitors. According to Blanc and Wolfe young duplicates (in this case, homeologous genes) would be more prone to recombine and disappear [36]. Therefore the unusual age profile of paralogous genes in tandem or in non-homeologous chromosomes indicates that their corresponding duplicated homeologous genes might have been deleted during the evolution. In short, duplications by allotetraploidy remained as the primary cause for the high number of *CCCH* genes in switchgrass and a high percentage of *CCCH* genes were under diversifying selection.

**Table 1:** **Purifying and diversifying selection of** ***PvC3Hs***

**Duplicated pairs**	**Chromosomal locations**	**Ks**	**Ka**	**Ka/Ks**	**Evolutionary Selection**	**Duplication type**	**Divergence Time (Mya)**
**PvC3H29/39**	Chr5a/5b	0.000	0.041	N/A	Diversifying	Homeologous	N/A
**PvC3H34/45**	Chr5a/5b	0.000	0.036	N/A	Diversifying	Homeologous	N/A
**PvC3H62/66**	Chr9a/9b	0.000	0.018	N/A	Diversifying	Homeologous	N/A
**PvC3H54/57**	Chr7a/7b	0.039	0.052	1.316	Diversifying	Homeologous	3
**PvC3H33/41**	Chr5a/5b	0.041	0.019	0.459	Purifying	Homeologous	3.1
**PvC3H6/7**	Chr1a/1b	0.046	0.075	1.649	Diversifying	Homeologous	3.5
**PvC3H61/65**	Chr9a/9b	0.047	0.03	0.638	Purifying	Homeologous	3.6
**PvC3H32/42**	Chr5a/5b	0.06	0.006	0.104	Purifying	Homeologous	4.6
**PvC3H35/44**	Chr5a/5b	0.065	0.044	0.672	Purifying	Homeologous	5
**PvC3H1/9**	Chr1a/1b	0.073	0.061	0.841	Purifying	Homeologous	5.6
**PvC3H31/43**	Chr5a/5b	0.076	0.066	0.874	Purifying	Homeologous	5.8
**PvC3H10/15**	Chr2a/2b	0.082	0.092	1.119	Diversifying	Homeologous	6.3
**PvC3H30/40**	Chr5a/5b	0.102	0.084	0.825	Purifying	Homeologous	7.9
**PvC3H20/23**	Chr3a/3b	0.131	0.064	0.489	Purifying	Homeologous	10.1
**PvC3H46/51**	Chr6a/6b	0.151	0.013	0.087	Purifying	Homeologous	11.6
**PvC3H5/6**	Chr1a/1a	0.163	0.06	0.367	Purifying	Tandem	12.5
**PvC3H17/50**	Chr3a/6b	0.237	0.03	0.128	Purifying	Paralogous	18.2
**PvC3H32/33**	Chr5a/5a	0.282	0.444	1.575	Diversifying	Tandem	21.7
**PvC3H41/42**	Chr5b/5b	0.287	0.464	1.616	Diversifying	Tandem	22.1

Ks: number of synonymous substitutions per synonymous site; Ka: number of nonsynonymous substitutions per nonsynonymous site. When Ka/Ks = 1, neutral evolution; Ka/Ks < 1, purifying selection; Ka/Ks > 1, diversifying selection. Genes in duplicated pairs are in tandem duplication (Tandem), in homeologous chromosomes (Homeologous) or were simply paralogous.

### Organ/tissue-level *PvC3H*s expression atlas discovered genes potentially involved in development of highly lignified cells and florets

**Figure 5:**
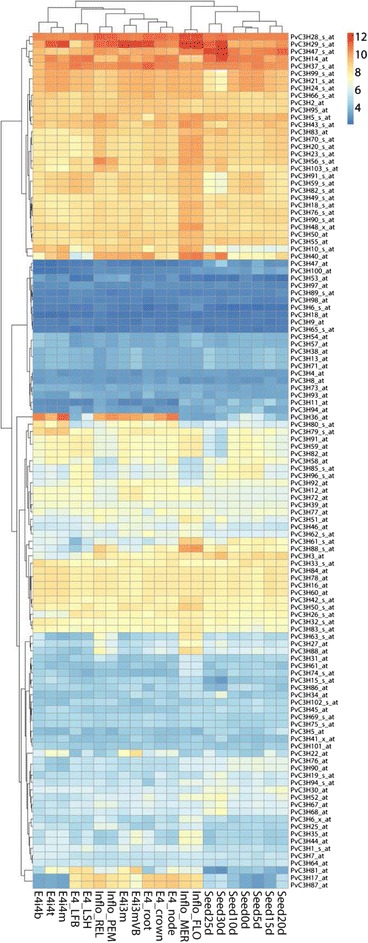
**Heatmap of expression levels of** ***PvC3H*** **s in 21 organs, tissues or at different developmental stages.** Abbreviations were adopted from PviGEA database as follows. *E4i4b*: Bottom 1/5 fragment of the 4th internode; *E4i4t*: Top 1/5 fragment of the 4th internode; *E4i4m*: Middle 1/5 fragment of the 4th internode 4; *E4-LFB*: Pooled leaf blade from plant ; *E4-LSH*: Pooled leaf sheath; *Inflo-REL*: Rachis and branch elongation of inflorescence (50–150 mm); *Inflo-PEM*: Panicle emergence of inflorescence (>200 mm); *E4i3m*: Middle 1/5 fragment of the 3rd internode; *E4i3m-VB*: Vascular bundle isolated from 1/5 fragment of the 3rd internode; *E4-root*: Whole root system; *E4-crown*: Whole crown; *E4-node*: Pooled nodes; *Inflo-MER*: Inflorescence meristem (0.5-3.0 mm); *Inflo-FLO*: Floret of inflorescence when glumes are 10–20 mm; *Seed0d*: Whole flowers at anthesis stage; *Seed5d*: Whole seeds 5 days post fertilization; *Seed10d*: Whole seeds with visible caryopsis; *Seed15d*: Whole seeds at the milk stage; *Seed20d*: Whole seeds at the soft dough stage; *Seed25d*: Whole seeds at the hard dough stage; *Seed30d*: Whole seeds at the physiological maturity stage.

**Figure 6:**
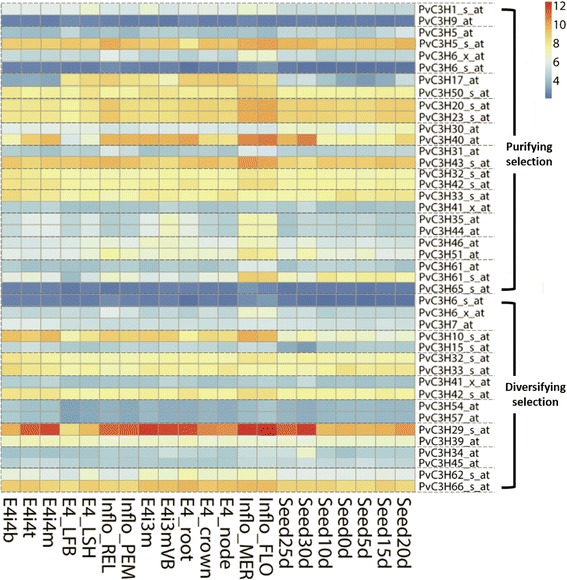
**Comparison of organ/tissue level expression atlas between paralogous** ***PvC3H*** **pairs under purifying and diversifying selection.**

The expression patterns of 103 *PvC3Hs* in 21 different organs, tissues and developmental stages were analyzed using data mined from the switchgrass Gene Expression Atlas (PviGEA) [23,24]. As shown in Figure 5, genes (represented by corresponding probes) and samples were clustered according to their corresponding expression patterns. Samples from vegetative organs/tissues and from reproductive organs were separated into two clusters. Notably, most probes detected relatively high transcripts levels with non-specific probes cross-hybridizing with a set of sequences (probes with an affix as _s_at) and mixed probe sets (_x_at) containing at least one probe that cross-hybridized with other sequences. Gene-specific probes (_at) detected that only seven *PvC3H* genes (*PvC3H14/2/95/83/50/55/40*) had relatively high expression levels in most organs/tissues. The gene expression atlas of 19 pairs of paralogous *PvC3Hs* were also compared (Figure 6). For the 11 pairs of *CCCH* genes under purifying selection, 8 pairs have similar organ/tissue-level expression patterns (73%); while for the rest of the 8 pairs under diversifying selection, only two pairs were similar (25%) (Figure 6).

The organ/tissue-level gene expression atlas is useful for predicting the functions of *PvC3H*s, especially for those potentially involved in plant development. For example, the *PvC3H36-*specific probe detected the gene only had high expression levels in lignified organs/tissues (e.g. node, internode, crown, roots and inflorescence branches), but not in less lignified tissues (e.g. leaf, leaf sheath, florets and seeds), suggesting the potential role of *PvC3H36* in the identity of lignified cells. Another interesting gene, *PvC3H22*, had expression levels in vascular bundles > nodes/internodes > leaf sheath > leaf blade, suggesting that this gene could be vascular cell-specific (Figure 5).

In another case, gene-specific probes detected that five genes (PvC3H40/88/74/35/44) had higher expression levels in florets and inflorescence meristems, suggesting that these five genes could be involved in switchgrass flower development. Interestingly, *PvC3H35* and −*44* were homologous to *AtHUA1* (Figures 3 and 5), reiterating their potential roles in regulating switchgrass flower development.

### Promoter and qRT-PCR analyses highlighted clade-XIV *PvC3H* gene*s* as ABA- and stress-responsive

**Figure 7:**
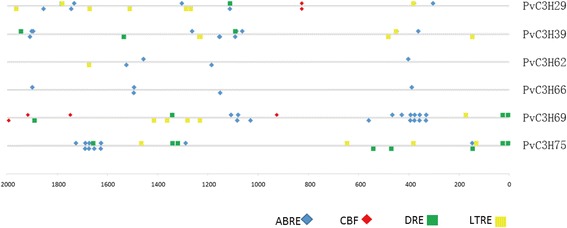
**Promoter analysis of six Clade-XIV** ***PvC3H*** **s.** Stress-related cis-elements of the −2 Kb 5′ upstream region of six *PvC3H*s were shown. Cis-elements in the sense-strand were indicated above the line, and those in the complementary-strand below the line.

We found that PvC3Hs in Clade-XIV were homologous to ABA- or stress-responsive *CCCH* genes in Arabidopsis and rice (Figure 3). We first performed a promoter analysis with six *PvC3H*s in Clade-XIV whose promoter sequences (−2.0 kb) were available in the switchgrass genome database [21] (Figure 7). Cis-elements, such as ABA Responsive Element (ABRE), Dehydration-Responsive Element (DRE), C-repeat Binding Factors (CBFHV), and Low Temperature Responsive Element (LTRE) of Clade-XIV genes’ promoters were shown in Figure 7. All six *PvC3H*s in Clade-XIV had multiple ABRE elements, and four *PvC3H*s had multiple DREs or CBFHVs/LTREs in their −2.0 kb promoter regions. The promoter analysis suggested that Clade-XIV genes should be responsive to ABA and stresses.

**Figure 8:**
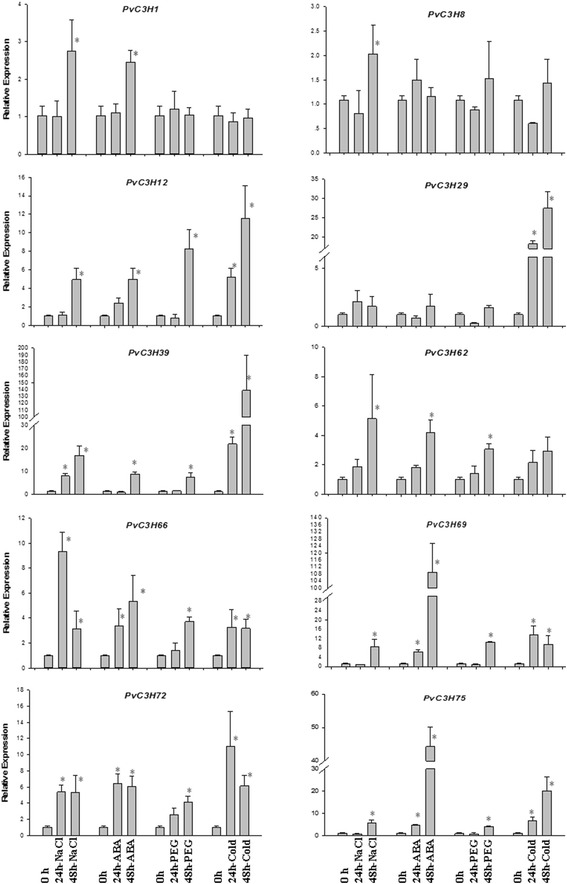
**qRT-PCR analysis of eight Clade-XIV PvC3Hs and two** ***PvC3H*** **s in the other clades.** Noting that *PvC3H1* and −*8* were chosen as controls for these two genes were not in Clade-XIV and their promoter regions had very few stress-responsive cis-elements. * Indicates statistically significant difference (P < 0.05) as compared with the control (0 h).

To validate this hypothesis, we carried out qRT-PCR with eight *PvC3H*s in Clade-XIV to see whether or not these genes were responsive to ABA and various stresses. Meanwhile, another two *PvC3H*s (*PvC3H1* & -*8*) with fewer ABREs and DREs in their promoters were picked as controls in the qRT-PCR experiment. Using the cut-off value of 2-fold change, we found that the transcript levels of all eight *PvC3H*s in Clade-XIV but not *PvC3H1* & -*8*, were dramatically induced under one or more stress treatments (Figure 8). In particular, the expression levels of *PvC3H29* and *PvC3H39*, orthologs to *OsDOS* (Figure 3), were drastically induced by cold treatment (27- and 138-fold changes, respectively). *PvC3H69* and *PvC3H75*, orthologs to *OsTZF1* (Figure 3), remarkably responded to ABA treatment (108- and 44-fold changes, respectively). The transcript level of *PvC3H66* increased to 9-fold after 24 hrs salt treatment and the transcript levels of *PvC3H12*/*62*/*72* increased to more than 5-fold after one or more of the stress treatments within 48 hrs.

## Discussion

### Large number of *CCCH* genes and higher percentage of them under diversifying selection reflect recent allopolyploidization event in tetraploid switchgrass

The estimated genome size of tetraploid switchgrass is ~1,600 Mb [37], which is smaller than that of maize (2,300 Mb) [38], but much bigger than that of rice (430 MB) [39,40]. The gene density in switchgrass was ~16.4 kb per gene [25], similar to rice (13.4 kb per gene) [39,40], but lower than maize (~35 kb per gene) [38]. Counting the genome size and average gene density, we can conclude that the number of genes in tetraploid switchgrass is ~1.5 times and ~3.0 times the number of genes in maize and rice, respectively. Consistently, the number of *CCCH* genes in switchgrass (103) is ~1.5 times that in maize (68). Yet, inconsistent with the above calculation, the number of *CCCH* genes in maize (68) and rice (67) are nearly the same.

The Poaceae family experienced a process of paleopolyploidization which happened around 70 Mya [41] and a subsequent “diploidization” process ~60 Mya [42]. Rice and the common ancestor of maize and sorghum diverged ~50 Mya [43]. Although maize genome went through additional whole-genome duplication ~5-12 Mya, at least 50% of its duplicated genes lost one or both member(s) over the past 5 million years [43]. More likely, the maize *CCCH* genes have undergone extensive gene loss or diversification process after the whole-genome duplication event, which ultimately lead to the current number of *CCCH* genes in maize.

**Figure 9:**
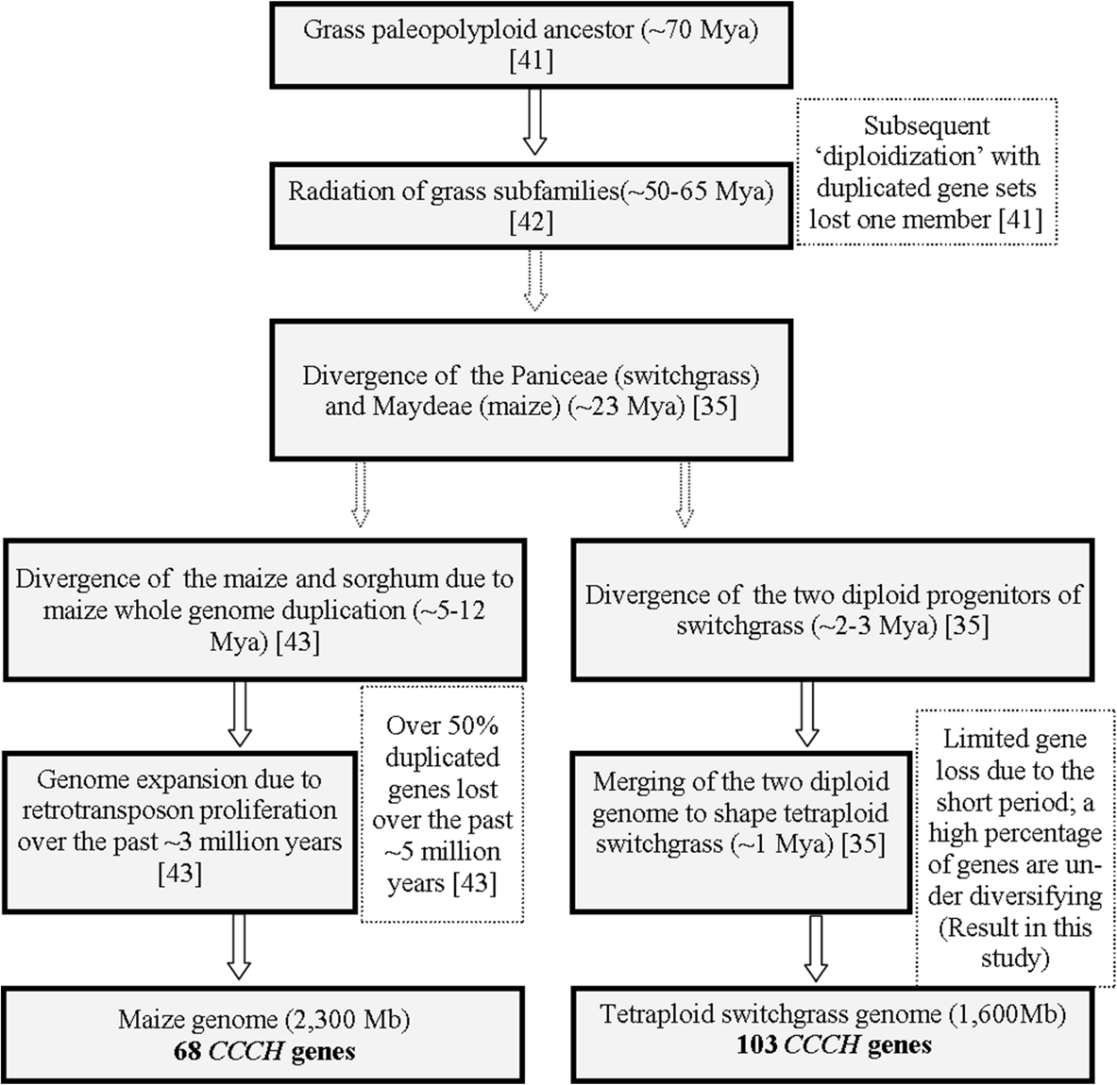
**Flow chart illustration of comparative evolutionary events in switchgrass and maize.**

The tribes Paniceae (switchgrass) and Maydeae (maize) diverged ~ 23 Mya [35]. It was proposed that the two sets of subgenomes of switchgrass originated from two closely related diploid progenitors which diverged less than 2 Mya, and the polyploidization events less than 1 Mya through comparing nucleotide substitution of the acetyl-coA carboxylase genes in homeologous chromosomes [35]. This estimation for the divergence time by Huang et al. [35] was largely consistent with our finding with *CCCH* genes that a large number of *PvC3H* gene pairs between homeologous chromosomes diverged ~3 Mya (Table 1). Meanwhile, 15 out of 16 pairs of paralogous *PvC3Hs* with defined chr. locations were on corresponding homeologous chromosomes, accounting for 93.8% of the gene duplication event, which confirms that the two sets of subgenomes were originated from closely-related diploid grasses. After the allotetraploidization event in switchgrass, we would expect a similar gene loss process which occurred during maize evolution. Yet, the short time window (1–2 million years) after polyploidization allowed the existence of redundant homeologous *CCCH* genes, even though a large percentage of them (42.1%) are under diversifying selection. Taken together, the recent divergence of switchgrass diploid progenitors and the polyploidization event sufficiently account for the higher number of *CCCH* genes in the tetraploid switchgrass genome. A flowchart was drawn for better illustration of the above reasoning (Figure 9).

Polyploidy is an important route for fast evolution in flowering plants [34]. Switchgrass is a self-incompatible out-crossing ployploid grass. According to its native growth habitats and phenotypic features, switchgrass was classified into lowland and upland ecotypes [18]. The lowland ecotypes were mainly tetraploid (2n = 4x = 36), while the upland varied from tetraploid, hexaploid (2n = 6x = 54) to octoploid (2n = 8x = 72) [25,35]. Associated with the allopolyploid genome arising from combinations of divergent diploids, the tetraploid switchgrass is disomic inheritance [35]. In contrast to polysomic inheritance, disomic inheritance in polyploids presents opportunities for duplicated genes to diverge and evolve new functions [34]. Consistent with this theory, we found 42.1% *CCCH* gene pairs were under diversifying selection in this study. A total of 14 *PvC3Hs* were potentially *Panicum*-specific (PvC3H1, −9, −15, −19, −22, −25, −48, −53, −61, −65, −74, −86, −89, and −97), for which no ortholog (bootstrap value >50) was found among maize, rice and Arabidopsis *CCCH* genes (Additional file 3). This result suggests that these newly evolved genes and genes under diversifying selection could have different functions which ultimately allowed the successful adaptation of switchgrass across a wide geographical area in the North America.

### *CCCH* gene family analysis facilitated functional gene discovery

CCCH type Znf proteins share an ancient origin which now can be found in both prokaryotes and eukaryotes. Plant *CCCH* genes play important roles in plant development, and abiotic and biotic stress responses. The order and logo of CCCH motifs, exon-intron structures, and presence and distribution of other functional domains in each clade were highly conserved, implying that genes in the same clade could have conserved or similar functions across di- and monocotyledonous plant species. Using the Blast2Go program [44], we listed the estimated functions of all *PvC3H* genes in Additional file 6.

Based on the phylogenetic analysis, uniform gene structure, conserved domains and genomic contexts, we established orthologous relationship between 18 well-characterized *CCCH* genes in model plants and switchgrass (Additional file 4). Most characterized Arabidopsis, rice and cotton *CCCH* genes are homologous to Clade-XIV *PvC3H*s which were ABA- and stress-responsive. Over-expressing AtOXS2, *AtSZF1*/2, *OsTZF1*, *AtTZF1*/*2*, and *GhZFP1* all lead to improved stress tolerance [9,10,13,14,45]. Overexpressing *OsTZF1* and *OsDOS* also delayed leaf senescence in rice [11,14]. *AtTZF4*/*5*/*6* and *OsGZF1* encoded functional proteins regulating embryo maturity and/or seed germination [16,46]. It is still unknown whether or not over-expressing *AtTZF4*/*5*/*6* and *OsGZF1* could improve plant stress tolerance, but gene expression data showed that at least *AtTZF4*/*5*/*6* were ABA responsive [16]. Therefore, it is safe to hypothesize that most *PvC3Hs* in Clade-XIV were ABA- and/or stress-responsive and potentially involved in plant stress signaling pathways. This hypothesis was further supported by the promoter and gene expression analyses.

We were able to find switchgrass orthologs to *OsC3H12*, *AtHUA1* and *OsLIC*. OsC3H12 quantitatively contribute to defense against bacterial pathogens in rice likely through the JA-dependent pathway [33]. OsC3H12 was homologous to Clade-I PvC3Hs with the distinct feature of five CCCH motifs with three at the N-terminal and two at the C-terminal. For switchgrass, pathogenic diseases (e.g. rust) are a potential threat if the bioenergy plant were grown in large scales. Identifying quantitative trait loci for disease resistance would be important for breeding switchgrass cultivars with long-term disease resistant trait.

Two switchgrass orthologs (*PvC3H27/68*) to *OsLIC* were found, which could be involved in the establishment of grass architecture. Overexpressing *OsLIC* in rice induced the ‘erect-leaf’ phenotype through reducing the leaf angle against the stem [47]. Small leaf angles (erect leaves) greatly contributed to a high leaf area index (LAI, ratio between upper leaf surface area and shaded land area) to increase light perception for photosynthesis, especially in dense planting field [48]. Switchgrass is a bunch-type grass with robust tillers but no rhizome or stolon. Therefore, reducing shading effect of the upper leaves through adjusting leaf angles should be a promising way to improve its biomass yield per unit of land area, and *PvC3H27/68* would be good candidate genes to work with for such purposes.

Two functional-annotated genes (*OsEhd4* and *AtFES1*) found no orthologs in switchgrass. *OsEhd4* encoded an Oryza-genus-specific regulator of photoperiod flowering in rice, which could be a rare *CCCH* gene resulted from diversifying selection. *AtFES1*, an Arabidopsis gene, was essential for the winter-annual habit of the herb by genetically suppressing FRI-mediated vernalization. Therefore, it was not surprising to see that the perennial grass switchgrass had no orthologs to *AtFES1*. This result was consistent with the gene duplication and divergence analysis. On the other hand, it would be interesting to see whether and how switchgrass-specific *CCCH* genes (e.g. *PvC3H19*/*22*) benefited the plants and shaped its unique plant statue and ecological fitness.

The functionality of the abovementioned *PvC3H* genes, particularly those orthologous to known functional rice and Arabidopsis *CCCH* genes, can be further confirmed through transgenic approaches. These *PvC3Hs* can be used in genetic engineering or as molecular markers in marker-assisted breeding to improve switchgrass agronomic traits, e.g., stress tolerance and delayed senescence.

## Conclusions

The genome-wide study of switchgrass *CCCH* genes determined phylogenetic classification, evolution, tissue/organ level gene expression, and potential functions of these genes. The large number of *CCCH* genes and high percentage of them under diversifying selection reflect the recent evolution events of allotetraploid switchgrass. The Clade-XIV*PvC3H*s were highlighted in this study for their responses to different abiotic stresses at transcriptional levels and for their potential regulatory roles in stress-tolerances. Manipulating the expression level of *CCCH* genes through biotechnological approaches could be an effective way to further improve the agronomic traits of switchgrass.

## Methods

### Identification and sequence analysis of CCCH proteins in switchgrass

The latest version (V1.1) of the switchgrass draft genome and protein sequences was downloaded from the phytozome database [21] to construct a local switchgrass protein database using HMMER software (http://hmmer.janelia.org) [49]. The Hidden Markov Model (HMM) file PF00642 (C–X_4–15_–C–X_4–6_–C–X_3_–H) for CCCHs was downloaded from Pfam (http://www.pfam.org) [50], which was used as a query to blast against the local database. All hits with E-values below 0.001 were selected and further confirmed by Pfam (PF00642) [50] and SMART (Sm00356) [51] to remove false positive sequences. All of confirmed CCCH proteins were aligned using ClustalX to manually remove the redundant sequences.

The number of CCCH motifs was counted using the EditPlus software by searching for the pattern of “C\w{i}\C\w{j}C\w{3}H” in which “i” ranged from 4 to 17 and “j” from 4 to 6. The conserved CCCH motifs were analyzed using Weblogo [52] for their sequence logos.

The neighbor-joining (N-J) phylogenetic tree for CCCH proteins of switchgrass, maize, rice and Arabidopsis was constructed using MEGA 6 with the alignment using ClustalX (bootstrap 1,000 replicates) [29]. For the 25 *PvC3H* genes with alternative splicing sites, we only picked their longest translated proteins in the CCCH motif and phylogenetic tree analysis to avoid duplicated result.

The chromosomal location, coding sequence (CDS), exons and introns number, ORF length and amino acid (AA) information of switchgrass *CCCH* genes was obtained from the phytozome database. The ExPASy program [53] was used to calculate CCCH proteins’ molecular weight (kDa) and isoelectric point (pI). Exon-intron display was constructed using the gene structure display server (http://gsds.cbi.pku.edu.cn) [54].

Chromosome location images were generated by using the MapInspect software to localize switchgrass *CCCH* genes. For those *CCCH* genes whose chromosome localization is unclear yet, we listed them in the right side in Figure 5. Tandem duplications of paralogous genes were defined as two paralogs separated by less than five genes in the same chromosome [7], segmental duplications were those placed on duplicated chromosomal blocks from the same genome lineage [7], while duplications in two sets of subgenomes (usually in homeologous chromosomes) can be explained by allotetraploidy (interspecific genome duplication) [55]. The ratio between nonsynonymous and synonymous nucleotide substitutions (Ka/Ks) was calculated using DNAsp5 software (http://www.ub.edu/dnasp/) [56] for selected pairs of homologous genes. The estimated divergence years of paired genes were calculated using the following equation: T = Ks/2λ × 10^−6^ (λ = 6.5 × 10^−9^ for grasses) [57].

The cis-element of selected *CCCH* genes promoter region (up to −2000 bp upstream of the CDS) were analyzed using the PLACE website (http://www.dna.affrc.go.jp/PLACE/) [58].

### Transcripts levels in 21 switchgrass organ/tissues and developmental stages

For the 103 identified *CCCH* genes in switchgrass, corresponding Unitranscript IDs were recognized for each gene in the PviUTs database [23,24]. The Unitranscript IDs were used to search against the integrated transcript sequence database, PviGEAs [23,24]. The resultant data from the database were graphically presented in a heatmap format as log_2_ fold change after value normalization using the R Project software.

### Plant material, growth condition and stress treatments

Switchgrass line ‘HR8’ [59] selected from the ecotype ‘Alamo’ was used to study the gene expression levels under various stress conditions. Switchgrass seeds were surface sterilized with 50% bleach for 30 min, washed 5 times with water, and sowed in sterilized medium containing peat moss: vermiculite (1:1). After 4 weeks of growth in a greenhouse [14 h photoperiod and 30/20 ± 3°C (day/night)] the plants were transplanted to half strength Hoagland solution with aeration for another 2 weeks before stress treatment. For stress treatments, the plantlets were cultured in 1/2 Hoagland solution containing 20% PEG, 250 mM NaCl or 100 μM ABA, or were placed in 4°C for cold treatment for 48 hours. The 2nd fully expanded leaves from the top of plantlets were collected for RNA preparation with three biological repeats per treatment.

### Real-time qRT-PCR

Total RNA was extracted using a column based RNA Extract kit (YPH-Bio Inc., Cat. No. HF103, Beijing, China), and treated with RNase-free DNaseI to eliminate gDNA (TaKaRa Biotech. Co. Ltd., DaLian, China). The RNA concentration and integrity were checked by spectrophotometry and gel electrophoresis. A total of 0.5 μg RNA per sample were reverse transcribed into cDNA with the PrimeScript^™^ II reverse transcription kit (TaKaRa). The cDNAs were diluted 1:10 with nuclease-free water prior to the qRT-PCR analyses.

qRT-PCR was performed with the LightCycler® 480 SYBRGreen I Master mix (Roche Ltd. Mannheim, Germany) using the Roche Light Cycler® 480 II Real-Time PCR System. The PCR reaction was performed in a 20 μl reaction volume following the manufacturer’s instructions. The data were normalized against the best rated reference genes, *PvFTSH4* [60] and *Actin2* [61]. Data presented were the averages of three biological repeats (samples). For each sample, two technical replicates were carried out in qRT-PCR analysis. The dissociation curves showed that primers used in the qRT-PCR were gene-specific (Additional file 7). The comparison of treatment means was analyzed by the Tukey HSD multiple comparison procedure using JMP software version 7 (SAS Inc., Cary NC).

## Additional files

Additional file: 1.
**Summary of PvC3Hs.** Proposed names of PvC3Hs in this study, gene identifier in the Phytozome database, chromosomal localization, length of ORF, molecular weight (MW), pI value were presented. Corresponding unitranscript ID in PviUTs and probesetID(s) in PviGEA were also shown in the table. Additional file: 2.
**Sequence logos for common Zf-CCCH motifs in switchgrass, maize, rice and Arabidopsis.** (A) C-X7-C-X5-C-X3-H motif; (B) C-X8-C-X5-C-X3-H motif. Additional file: 3.
**Phylogenetic relationships of switchgrass, maize, rice and Arabidopsis CCCH proteins.** The evolutionary history was inferred using the Neighbor-Joining method in MEGA6, with bootstrap test set at 1000 replicates. Bootstrap values of 1,000 replications were executed, and only results above 50 are shown at each node. Additional file: 4.
**Plant functional-annotated** ***CCCH*** **genes and corresponding orthologs of** ***PvC3H*** **s.**
Additional file: 5.
**Microsyntenic regions between tandem duplicated** ***CCCH*** **genes in switchgrass and maize.** Red bars indicate tandem duplications. Purple dashed lines indicate corresponding syntenic gene sets in the two species which result was supported by phylogenetic analysis in Additional file 2. The maize *CCCH* chromosome location map was revised from Peng et al. (2012) [20]. Additional file: 6.
**Gene functional annotation for** ***CCCH*** **genes in switchgrass using Blast2GO program.**
Additional file: 7.
**Primers used in qRT-PCR.**

## Abbreviations

Aa: Amino acid
Ank: Ankyrin repeat
BLAST: The Basic Local Alignment Search Tool
CDS: Coding sequence
HMM: Hidden Markov Model
KH: K homolog domain
kD: Kilo-dalton
MW: Molecular weight
Mya: Million years ago
N-J: The neighbor-joining
Pfam: Protein family
pIs: Isoelectric points
qRT-PCR: Quantitative real-time PCR
RRM: RNA-Recognition Motif
WD40: WD40 repeat
Znf: Zinc finger
