# Supplementary figures and images for: Comprehensive analysis of CCCH-type zinc finger family genes facilitates functional gene discovery and reflects recent allopolyploidization event in tetraploid switchgrass
Source: BMC Genomics. 2015 Feb 25;16(1):129. doi: 10.1186/s12864-015-1328-4 (PMC4352264; doi:10.1186/s12864-015-1328-4)

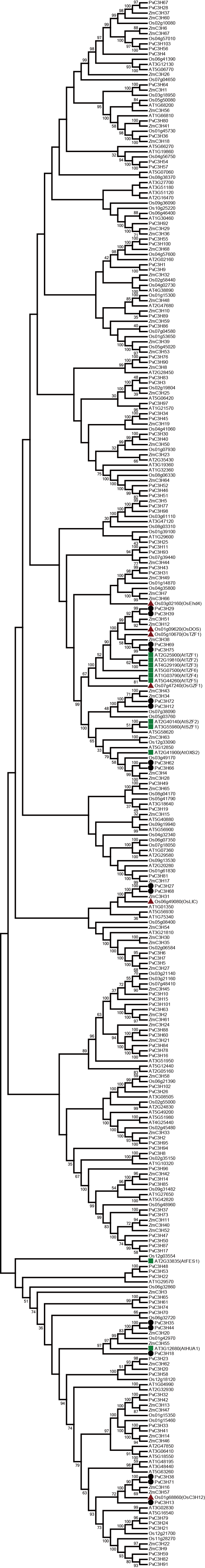

Supplement: Additional file: 3. — Phylogenetic relationships of switchgrass, maize, rice and Arabidopsis CCCH proteins. The evolutionary history was inferred using the Neighbor-Joining method in MEGA6, with bootstrap test set at 1000 replicates. Bootstrap values of 1,000 replications were executed, and only results above 50 are shown at each node. [file 12864_2015_1328_MOESM3_ESM.png]

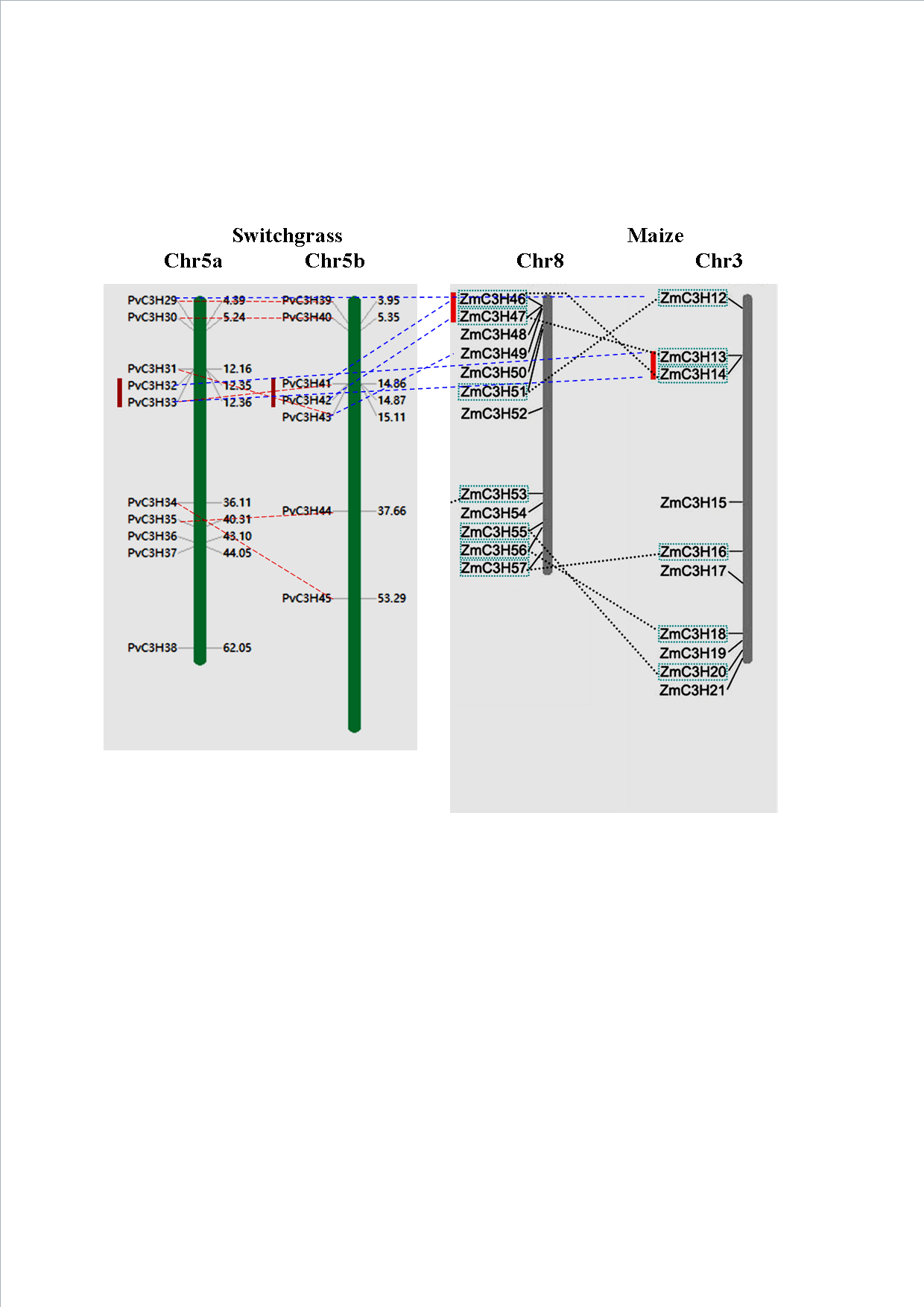

Supplement: Additional file: 5. — Microsyntenic regions between tandem duplicated CCCH genes in switchgrass and maize. Red bars indicate tandem duplications. Purple dashed lines indicate corresponding syntenic gene sets in the two species which result was supported by phylogenetic analysis in Additional file 2. The maize CCCH chromosome location map was revised from Peng et al. (2012) [20]. [file 12864_2015_1328_MOESM5_ESM.png]
