# Supplementary material for: Comprehensive analysis of CCCH-type zinc finger family genes facilitates functional gene discovery and reflects recent allopolyploidization event in tetraploid switchgrass
Source: BMC Genomics. 2015 Feb 25;16(1):129. doi: 10.1186/s12864-015-1328-4 (PMC4352264; doi:10.1186/s12864-015-1328-4)
Supplement: Additional file: 4. — Plant functional-annotated CCCH genes and corresponding orthologs of PvC3H s. [file 12864_2015_1328_MOESM4_ESM.doc]

**Additional file 4. Plant functional-annotated CCCH genes and corresponding orthologs of *PvC3H*s**

| **CCCH name** | **Locus name** | **Gene functional annotation** | **References** | **Closest PvC3H homolog(s)** |
| --- | --- | --- | --- | --- |
| OsC3H12 | Os01g68860 | Quantitatively contribute to the bacterial resistance, likely through JA-dependent pathway | Deng et al., 2012 | PvC3H38/71 in Clade- I |
| AtHUA1 | AT3G12680 | Floral development through interacting with AGAMOUS | Li et al., 2001 ; Cheng et al., 2003 | PvC3H18/35/44 in Clade-I |
| OsLIC | Os06g49080 | Mediate rice architecture via BR signaling | Wang et al., 2008 ; Zhang et al., 2012 | PvC3H27/68 in Clade-IX |
| OsEhd4 | Os03g02160 | Involved in photoperiodic control of flowering time. | Gao et al., 2013 | None |
| AtFES1 | AT2G33835 | Required for the winter-annual habit of Arabidopsis | Schmitz et al., 2005 | None |
| AtOXS2 | AT2G41900 | Flowering and stress integrator, responsive to ABA, cold, Cd and osmotic stresses. | Blanvillain et al., 2011 | PvC3H62/66 in Clade- XIV |
| AtSZF1 | AT3G55980 | Regulate salt stress response through transcriptional activating ethylene mediated signaling pathway | Sun et al., 2007 | PvC3H12/72 in Clade- XIV |
| AtSZF2 | AT2G40140 | Similar to AtSZF1; Overexpressing AtSZF2 causes more salt tolerance and a reduced expression of many salt-responsive genes | Sun et al., 2007 | PvC3H12/72 in Clade- XIV |
| OsDOS | Os01g09620 | Delay leaf senescence possibly through JA pathway; | Kong et al., 2006 | PvC3H39/29in Clade-XIV |
| OsTZF1 | Os05g10670 | Delayed senescence and enhanced stress tolerance possibly through regulating GA and ABA | Jan et al. 2013 Pomeranz et al., 2011 | PvC3H75/69 in Clade-XIV |
| AtTZF1/ AtCTH | AT2G25900 | Affects ABA-,GA-mediated growth and stress responses | Lin et al., 2011 | PvC3H39/29/75/69 in Clade-XIV |
| AtTZF2/ AtOZF1 | AT2G19810 | Similar to TZF3; involved in ABA/JA responses | Lee et al., 2012 | PvC3H39/29/75/69 in Clade-XIV |
| AtTZF3/ AtOZF2 | AT4G29190 | Involved in ABA/ JA/ salt stress responses through ABI2-mediated signaling pathway | Lee et al., 2012 | PvC3H39/29/75/69 in Clade-XIV |
| AtTZF4/ SOMNUS | AT1G03790 | Negatively regulate light-dependent seed germination; up-regulated by ABA and down-regulated by GA | Bogamuwa & Jang, 2013 | PvC3H39/29/75/69 in Clade-XIV |
| AtTZF5 | AT5G44260 | Similar to AtTZF4, involved in light-, ABA- and GA-mediated seed germination | Bogamuwa & Jang, 2013 | PvC3H39/29/75/69 in Clade-XIV |
| AtTZF6/ PEI1 | AT5G07500 | Similar to AtTZF4&5; also required for heart-stage embryo formation; | Li et al., 1998 ; Bogamuwa & Jang, 2013 | PvC3H39/29/75/69 in Clade-XIV |
| OsGZF1 | Os07g47240 | Rice seed N-storage regulator, directly bind the GluB-1 gene’s promoter. | Chen et al., 2014 | None |
| GhZFP1 | AY887895 | Cotton CCCH gene conferring salt and fungal-disease resistance. | Guo et al., 2009 | PvC3H39/29/75/69 in Clade-XIV |
| MsZFN | EU624138.1 | When ectopically overexpressed in Arabidopsis, it postponed Arabidopsis flowering time | Chao et al., 2014 | PvC3H38/71 in Clade-I |

**References:**

1. Deng H, Liu H, Li X, Xiao J, Wang S: **A CCCH-type zinc finger nucleic acid-binding protein quantitatively confers resistance against rice bacterial blight disease**. *Plant Physiol* 2012, **158**(2):876-889.

2. Li J, Jia D, Chen X: **HUA1, a regulator of stamen and carpel identities in Arabidopsis, codes for a nuclear RNA binding protein**. *Plant Cell* 2001, **13**(10):2269-2281.

3. Cheng Y, Kato N, Wang W, Li J, Chen X: **Two RNA binding proteins, HEN4 and HUA1, act in the processing of AGAMOUS pre-mRNA in *Arabidopsis thaliana***. *Dev Cell* 2003, **4**(1):53-66.

4. Wang L, Xu Y, Zhang C, Ma Q, Joo S-H, Kim S-K, Xu Z, Chong K: **OsLIC, a novel CCCH-type zinc finger protein with transcription activation, mediates rice architecture via brassinosteroids signaling**. *PloS one* 2008, **3**(10):e3521.

5. Zhang C, Xu Y, Guo S, Zhu J, Huan Q, Liu H, Wang L, Luo G, Wang X, Chong K: **Dynamics of brassinosteroid response modulated by negative regulator LIC in rice**. *PLoS Genet* 2012, **8**(4):e1002686.

6. Gao H, Zheng XM, Fei G, Chen J, Jin M, Ren Y, Wu W, Zhou K, Sheng P, Zhou F *et al*: **Ehd4 encodes a novel and *Oryza*-genus-specific regulator of photoperiodic flowering in rice**. *PLoS Genet* 2013, **9**(2):21.

7. Schmitz RJ, Hong L, Michaels S, Amasino RM: **FRIGIDA-ESSENTIAL 1 interacts genetically with FRIGIDA and FRIGIDA-LIKE 1 to promote the winter-annual habit of *Arabidopsis thaliana***. *Development* 2005, **132**(24):5471-5478.

8. Blanvillain R, Wei S, Wei P, Kim JH, Ow DW: **Stress tolerance to stress escape in plants: role of the OXS2 zinc-finger transcription factor family**. *Embo J* 2011, **30**(18):3812-3822.

9. Sun J, Jiang H, Xu Y, Li H, Wu X, Xie Q, Li C: **The CCCH-type zinc finger proteins AtSZF1 and AtSZF2 regulate salt stress responses in Arabidopsis**. *Plant Cell Physiol* 2007, **48**(8):1148-1158.

10. Kong Z, Li M, Yang W, Xu W, Xue Y: **A novel nuclear-localized CCCH-type zinc finger protein, OsDOS, is involved in delaying leaf senescence in rice**. *Plant Physiol* 2006, **141**(4):1376-1388.

11. Jan A. MK, Todaka D., Kidokoro S., Abo M., Yoshimura E., Shinozaki K., Nakashima K., Yamaguchi-Shinozaki K.: **OsTZF1, a CCCH-tandem zinc finger protein, confers delayed senescence and stress tolerance in rice by regulating stress-related genes**. *Plant Physiol* 2013, **161**:1202-1216.

12. Pomeranz M, Finer J, Jang JC: **Putative molecular mechanisms underlying tandem CCCH zinc finger protein mediated plant growth, stress, and gene expression responses**. *Plant Signal Behav* 2011, **6**(5):647-651.

13. Lin PC, Pomeranz MC, Jikumaru Y, Kang SG, Hah C, Fujioka S, Kamiya Y, Jang JC: **The Arabidopsis tandem zinc finger protein AtTZF1 affects ABA- and GA-mediated growth, stress and gene expression responses**. *Plant J* 2011, **65**(2):253-268.

14. Lee SJ, Jung HJ, Kang H, Kim SY: **Arabidopsis zinc finger proteins AtC3H49/AtTZF3 and AtC3H20/AtTZF2 are involved in ABA and JA responses**. *Plant Cell Physiol* 2012, **53**(4):673-686.

15. Bogamuwa S, JANG JC: **The Arabidopsis tandem CCCH zinc finger proteins AtTZF4, 5 and 6 are involved in light-, abscisic acid- and gibberellic acid-mediated regulation of seed germination**. *Plant Cell Environ* 2013, **36**(8):1507-1519.

16. Li Z, Thomas TL: **PEI1, an embryo-specific zinc finger protein gene required for heart-stage embryo formation in Arabidopsis**. *Plant Cell* 1998, **10**(3):383-398.

17. Chen Y, Sun A, Wang M, Zhu Z, Ouwerkerk PF: **Functions of the CCCH type zinc finger protein OsGZF1 in regulation of the seed storage protein GluB-1 from rice**. *Plant Mol Biol* 2014, **84**(6):621-634.

18. Guo YH, Yu YP, Wang D, Wu CA, Yang GD, Huang JG, Zheng CC: **GhZFP1, a novel CCCH-type zinc finger protein from cotton, enhances salt stress tolerance and fungal disease resistance in transgenic tobacco by interacting with GZIRD21A and GZIPR5**. *New Phytol* 2009, **183**(1):62-75.

19. Chao Y, Zhang T, Yang Q, Kang J, Sun Y, Gruber MY, Qin Z: **Expression of the alfalfa CCCH-type zinc finger protein gene MsZFN delays flowering time in transgenic Arabidopsis thaliana**. *Plant Sci* 2014, **215–216**(0):92-99.
