# Supplementary material for: Comprehensive analysis of CCCH-type zinc finger family genes facilitates functional gene discovery and reflects recent allopolyploidization event in tetraploid switchgrass
Source: BMC Genomics. 2015 Feb 25;16(1):129. doi: 10.1186/s12864-015-1328-4 (PMC4352264; doi:10.1186/s12864-015-1328-4)
Supplement: Additional file: 7. — Primers used in qRT-PCR. [file 12864_2015_1328_MOESM7_ESM.doc]

**Additional file 7. Primers used in qRT-PCR**

| **Target gene** | **Primer Name** | **Primer sequence (5’ to 3’)** |
| --- | --- | --- |
| **PvFTSH4** | PvFTSH4_F | TGGATGGCTTTAAGCAGAATGA |
|  | PvFTSH4_R | CAAAACGCCCAGGTCTGACT |
| **PvACT2** | PvACT2_qRT_F | GCGAGCTTCCCTGTAGGTA |
|  | PvACT2_qRT_R | CGAACCCAGCCTTCACCATAC |
| **Pavir.Aa00025** | P.Aa00025_F | TCAGGATGTAGAGGCAGCAG |
|  | P.Aa00025_R | GTCGTCCTGGTACTCCTGGT |
| **Pavir.Ab03367** | P.Ab03367_F | GAGGAGAACATGGAGGGAAA |
|  | P.Ab03367_R | AGCCGCCTCGTAGTCATAGT |
| **Pavir.Ab01939** | P.Ab01939_F | AAGGAACACAGCAGCACAAG |
|  | P.Ab01939_R | TGTAGCAACCCACCTGTCAT |
| **Pavir.Ba00634** | P.Ba00634_F | TTCAATGCAAGAGATGCACA |
|  | P.Ba00634_R | CGAATGCTCAGACCAGAAGA |
| **Pavir.Ea00362** | P.Ea00362_F | AGCCGCTTGATGTCTCCTT |
|  | P.Ea00362_R | AGCCTCTCGAACACCCTCT |
| **Pavir.Ga01774** | P.Ga01774_F | TTGGAGTACCAATGATGGGA |
|  | P.Ga01774_R | GTATGTGGATCAGGGTGCAA |
| **Pavir.Ga00204** | P.Ga00204_F | GAGGTTGAAGATGCTGCTGA |
|  | P.Ga00204_R | AGTCATCTGACTCGCCCTCT |
| **Pavir.Ia01456** | P.Ia01456_F | ATCGTCCTTTGAGCTGAGGT |
|  | P.Ia01456_R | GGTGAGGGTCCAACAGAGTT |
| **Pavir.Eb00253** | P.Eb00253_F | CGCTTGATGTCTCCTTCATC |
|  | P.Eb00253_R | AGCCTCTCGAAGACCTTC |
